# Finding disagreement pathway signatures and constructing an ensemble model for cancer classification

**DOI:** 10.1038/s41598-017-10258-5

**Published:** 2017-08-30

**Authors:** Qiaosheng Zhang, Jie Li, Dong Wang, Yadong Wang

**Affiliations:** 1 Harbin Institute of Technology, School of Computer Science and Technology, Harbin, 150001 P.R. China; 2 Heilongjiang Bayi Agricultural University, College of Science, Daqing, 163319 P.R. China

## Abstract

Cancer classification based on molecular level is a relatively routine research procedure with advances in high-throughput molecular profiling techniques. However, the number of genes typically far exceeds the number of the sample size in gene expression studies. The existing gene selection methods are almost based on statistics and machine learning, overlooking relevant biological principles or knowledge while working with biological data. Here, we propose a robust ensemble learning paradigm, which incorporates multiple pathways information, to predict cancer classification. We compare the proposed method with other methods, such as Elastic SCAD and PPDMF, and estimate the classification performance. The results show that the proposed method has the higher performances on most metrics and robust performance. We further investigate the biological mechanism of the ensemble feature genes. The results demonstrate that the ensemble feature genes are associated with drug targets/clinically-relevant cancer. In addition, some core biological pathways and biological process underlying clinically-relevant phenotypes are identified by function annotation. Overall, our research can provide a new perspective for the further study of molecular activities and manifestations of cancer.

## Introduction

For the patient to receive appropriate therapy, accurate classification of cancer is crucial in disease treatment^1, 2^. Accurate classification of cancer is the initial and significant step for clinical management since different treatment modalities exist. Traditionally, the classification of cancer is primarily based on the experience or histology. With advances in high-throughput sequencing techniques, the researchers can utilize the expression of tens of thousands of genes simultaneously. Cancer classification based on molecular level is now a relatively routine research procedure. However, the number of genes typically far exceeds the number of the sample size in gene expression studies. This situation is called high-dimensional and low sample size problem^3^.

To address the problem of high dimensionality, gene selection is one of the important steps for classification modeling. Gene selection is of fundamental and practical interest. To date, many types of gene selection methods were proposed. Guyon *et al*.^4^ proposed a SVM method of Recursive Feature Elimination (RFE) to gene selection by measuring the relative contribution of a gene. Li *et al*.^5^ employed maximum relevance minimum redundancy (mRMR) method based on Random Forest algorithm (RF) to predict protein cleavage sites. In Cai *et al*.’ work^6^ the authors performed ensemble-based feature extraction method, which incorporates Multi-category Receiver Operating Characteristic (Multi-ROC), Random Forests (RFs) as well as Maximum Relevance and Minimum Redundancy (mRMR) methods, to select molecular signatures. For gene selection, an alternative technique is the regularization method, such as lasso^7^ 1-norm support vector machine^8^ SCAD^9^ Elastic Net^10^ and Elastic SCAD^11^.

However, the above-mentioned gene selection methods are based on statistics and machine learning, seldom do these methods involve relevant biological principles or knowledge while working with biological data. So, many gene selection methods are prone to over-fitting or poor biological interpretation when applied on biological high-dimensional data. To improve the discriminant capability of features, biological domain knowledge, such as pathways are more often referred to during cancer classification to give more robust and generalizable results^12–19^. Pathways, being a series of interactions among molecules (including genes, gene products and compounds etc.), yield stable sets of functional relationships related with molecular biological activities such as metabolic, signaling, protein interaction and gene regulation processes, which plays an important role in understanding the mechanisms of complex diseases, improving clinical treatment, discovering drug target and biomarker^20^. Pathway-based method not only reduces the number of dimensions and increases statistical power, but also helps scientists better understand biological mechanisms at the molecular level^21^. For example, Kim *et al*.^19^ proposed standardized pathway-based approach extracting multi-level hierarchical feature vectors, with a basic gene level as well as a second level of pathway markers, to biomarker analysis for discriminating cancer subtypes. Huang *et al*.^17^ developed a personalized pathway-based diagnostic modeling framework(abbreviated as PPDMF) which converts omics-level features to pathway-level features using the non-parametric principle curve approach and subjects them to feature selection and machine learning classifications for differentiating different phenotypes.

**Figure 1:**
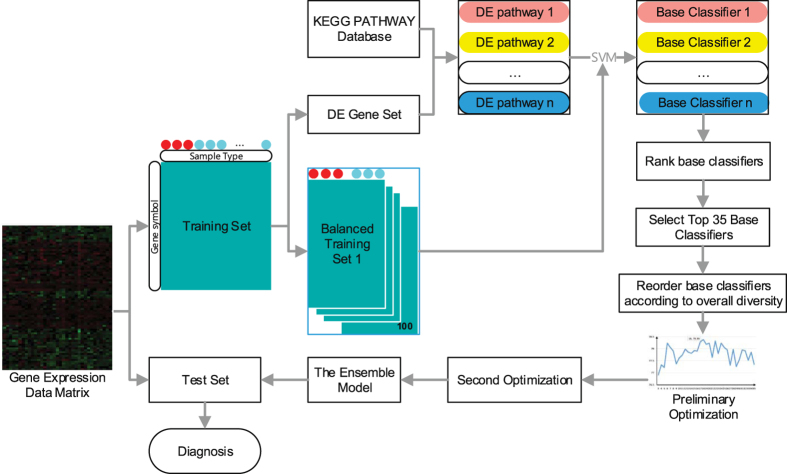
Overview of the proposed method. Dataset is randomly split into two separate groups, half for training and half for testing. The gene set of DE pathway is selected as features to train a certain SVM as a base classifier. The performance of each pathway-based classifier is tested on balanced training set using 5 fold bootstrap cross-validation with 100 runs (100 × 5). Then, we rank base classifiers according to average accuracies and calculate the diversity matrix between top 35. Base classifiers are reordered according to overall diversity. Preliminary optimization: Base classifier is added into the ensemble one by one in each iteration step from top 35, the highest accuracy of the ensemble with *m* base classifiers was obtained. Second optimization: classifiers selection is made by taking both accuracy and diversity into account from *m* base classifiers. Finally, the remaining base classifiers are combined as ensembles.

What distinguishes this work from the above is our goal to construct an ensemble learning framework, which incorporates pathway information, to predict cancer classification. Firstly, screening of differentially expressed(DE) genes is performed on training set of gene expression profiles. We select differentially expressed genes of each pathway to generate a group of base learners through training SVM, then, we rank all DE pathways with classification accuracy on training set. Secondly, the diversities of top 35 pathway-based base learners with higher accuracy are computed. Selecting classifiers into the ensemble from the top 35 pathway-based base classifiers according to diversity(for details see algorithm 1). Finally, integrating the remaining classifiers into the final ensemble learning model^22^ (see Fig. 1). Ensemble approach uses the final model in their decision making on testing dataset. Experimental results on different data sets in this paper indicate that our proposed method is very promising and robust.

## Materials and Methods

### Data

To evaluate the predictive ability of the here presented model, three publicly available gene microarray datasets are used to carry out analysis. For dataset GSE25066^23^ it is available via the Gene Expression Omnibus (ID = GSE25066) and includes 488 samples of breast cancer patients treated with NAC (antracyclines/taxanes) profiled with the U133A microarray. This dataset compared 99 pathologic complete response (pCR) samples and 389 residual disease (RD) samples (http://www.ncbi.nlm.nih.gov/geo/query/acc.cgi?acc=GSE25066). For dataset Liver^24^ it is one RNA-Seq data set from The Cancer Genome Atlas (TCGA) (https://gdc-portal.nci.nih.gov/legacy-archive/search/f). The Liver dataset consists of 421 samples obtained from comparing 371 liver cancer samples with 50 normal samples using the Agilent platform. For dataset GSE20194^25^ it is also a chemotherapy response data and comes from the Gene Expression Omnibus (ID = GSE20194). This dataset compared 56 pathologic complete response (pCR) samples and 222 residual disease (RD) samples (https://www.ncbi.nlm.nih.gov/geo/query/acc.cgi?acc=GSE20194). Pathways are come from KEGG database (http://www.genome.jp/kegg/). The total number of known human pathways in the KEGG database is 307. We selected 298 pathways of these containing at least one gene.

### Calculate differentially expressed genes

Differentially expressed genes between different phenotypes are thought to be fertile sources of stable cancer biomarkers. Hence, filtering out genes that are differentially expressed between different phenotypes is an integral part of understanding the molecular basis of phenotypic variation in cancers^26^. In the paper, we performed an exact test on training set of gene expression profile data to find genes that are differentially expressed between different phenotypes. Genes are considered to be significantly differentially expressed if they obtain a p-value <0.05. Then, we obtained a list of genes that are differentially expressed from gene expression profile data. The next step was to map each pathway to the list of differentially expressed genes (called as DE pathway).

### Rank base classifiers according to classification accuracy

The differentially expressed genes of each pathway were selected as classification features for each base classifier, respectively. Since Support Vector Machine (SVM) has been successfully applied to cancer classification using gene expression data^27^ we took the selected feature sets as input and used SVM as base classifier to discriminate between the two classes of interest. In order to form a baseline measure, we used default parameter settings for all SVM tasks. Next, all experiments were repeated for 100 runs on training set of gene expression profile data and the average accuracy of each base classifier was computed as the final results. Finally, base classifiers were ranked in descending order according to accuracy.

### Calculate diversity of base classifiers

It is well known that diversity among base classifiers plays an important role in ensemble learning. Ensembles tend to yield better results when there is a significant diversity among the base classifiers. There exist many measures of dependency between classifiers. The most commonly employed traditional measures of diversity adopt the zero-one loss (classification error) function, one of which is the disagreement measure. The disagreement measure estimates the diversity for a pair of classifiers in a form of a ratio between the number of samples for which classifiers disagreed, to the total number of observations. We carried out our work based on the disagreement measure due to its easy interpretation for independence, positive/negative dependences, and calculation^28^.

Let *h*_*i*_, *h*_*k*_ represents two different base classifiers, respectively. *L* = {*l*_1_, *l*_2_, …, *l*_n_} be a labeled data set. ${y}_{i}={\{{y}_{\mathrm{1,}i},{y}_{\mathrm{2,}i},\ldots ,{y}_{n,i}\}}^{T}$ represents the output of a base classifier *h*_*i*_, such that ${y}_{j,i}=1$, if *h*_*i*_ recognizes correctly *l*_*j*_, 0 otherwise. The diversity between two binary classifier outputs (correct/incorrect) *h*_*i*_, *h*_*k*_ is

1 $${D}_{i,k}=\frac{{N}^{01}+{N}^{10}}{{N}^{11}+{N}^{10}+{N}^{01}+{N}^{00}}$$

where *N* ^*ab*^ is the number of elements *l*_*j*_ of *L* for which ${y}_{j,i}=a$ and ${y}_{j,k}=b$ (see Table 1).

**Table 1:** Relationship between a pair of classifiers.

	*D*_*j*_ correct (1)	*D*_*j*_ correct (0)
*D*_*j*_ correct (1)	*N* ^11^	*N* ^10^
*D*_*j*_ correct (0)	*N* ^01^	*N* ^00^

The diversities of top *N* base-learners with higher accuracy were computed by formula (1). Finally, a diversity matrix *D* with *N* rows and *N* columns, which is symmetric was obtained.

### Optimize the ensemble based on diversity

**Figure 2:**
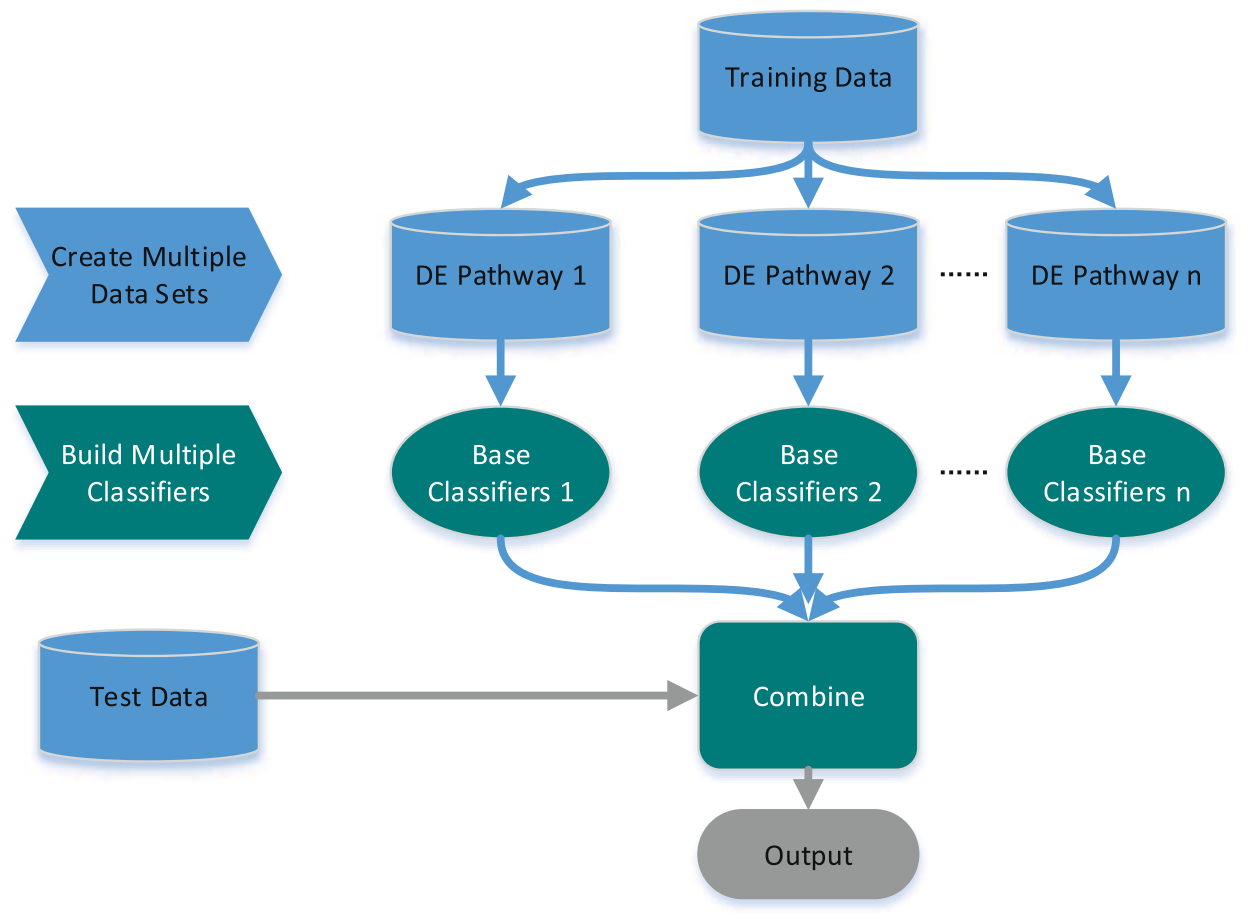
The framework of ensemble learning.

In the present study, genes of each DE pathway were used as features to train a certain SVM as a base learner. Since diversity plays an important role in ensemble learning, optimal selection of base classifiers was made by taking both accuracy and diversity into account (for the pseudo-code see algorithm 1). Finally, the ensemble classifier was constructed by selected base learners (see Fig. 2).

Let *S* denotes ordered DE pathway sets corresponding to feature sets of top *N* base learners based on DE pathways with higher accuracy. In order to reduce the computational complexity, we defined overall diversity of the *i* th pathway-based base classifier as *OD*[*i*]. *OD*[*i*] was calculated as follow:

2 $$OD[i]={\sum }_{j}(D[i,j]+D[j,i])$$

where *D* is a diversity matrix, $i\in 1\cdots N$.

Among different voting strategies, the majority voting is considered as a simplest and effective scheme^29, 30^. A majority vote based classifier ensemble technique classifies a pattern by letting each member of the ensemble cast a single vote for the correct class and deciding according to democratic rules. In the paper, we combined different base classifiers based on DE pathways and used a majority vote rule. The ensemble decision will be correct if at least $\lfloor \frac{T}{2}+1\rfloor$ classifiers choose the correct class, where *T* denotes the number of base classifiers. Firstly, we calculated *OD*[*i*] of base classifier from *S* and reordered base classifiers of *S* as *S* ^*^ in descending order according to *OD*. Secondly, base classifier based on DE pathway was incrementally added into the ensemble one by one in each iteration step from *S* ^*^. In each iteration, the average accuracy of each ensemble learning was obtained using 5-fold bootstrap cross-validation with 100 runs (100 × 5).Then, the highest accuracy of the ensemble with *m* base classifiers was obtained. Let *S*′ denotes ordered DE pathway set corresponding to top m base learners according to *OD*. Finally, the ensemble with m base classifiers was optimized according to algorithm 1. If the diversity of two base classifiers is smaller than the diversity threshold value *θ*, we think one of two base classifiers (or even two) is superfluous. Let *S* ^1^ denotes that two base classifiers are not removed from *S*′, *S* ^2^ denotes that one of the two base classifiers is removed from *S*′, *S* ^3^ denotes that another one of the two base classifiers is removed from *S*′, *S* ^4^ denotes that both of them are removed from *S*′. Then, we determined whether it can be removed through average classification accuracy obtained using 5-fold bootstrap cross-validation with 100 runs (100 × 5). When the classification accuracy is equal, the priority option is *S* ^4^ > *S* ^3^ > *S* ^2^ > *S* ^1^. The procedure was repeated until all base classifiers which can be deleted were removed, and the optimized ensemble with base learners from *S*″ was finally obtained.

## Results

### Classification performance of the proposed ensemble method

To verify our method, we conducted computational experiments on Dataset GSE25066. We evaluated the performance of the proposed ensemble method through five measures: accuracy, precision (Positive Predictive Value), sensitivity (True Positive Rate), specificity and F-score which are calculated below:
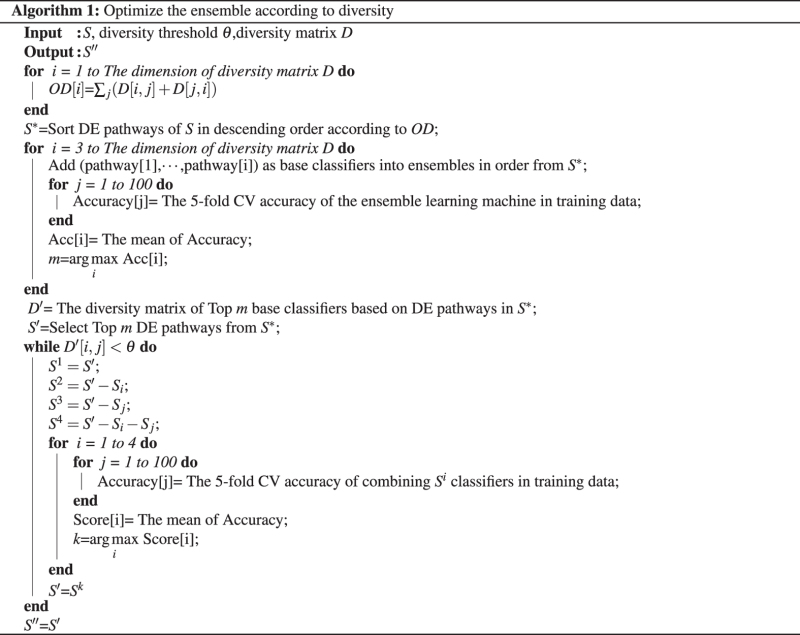

3 $$\{\begin{array}{rcl}accuracy & = & \frac{TP+TN}{TP+TN+FP+FN}\\ precision & = & \frac{TP}{TP+FP}\\ sensitivity & = & \frac{TP}{TP+FN}\\ specificity & = & \frac{TN}{TN+FP}\\ F-score & = & \frac{2\times TP}{2TP+FP+FN}\end{array}$$

where TP denotes true positive, TN denotes true negative, FP denotes false positive and FN denotes false negative. Firstly, the GSE25066 dataset was randomly split into two separate groups according to sample types, half for training (50 pCR vs. 195 RD) and half for testing (49 pCR vs. 194 RD). Limma^31^ is an R/Bioconductor software package that allows users to analyse both RNA-seq and microarray data with very similar pipelines. Among the methods evaluated for differential expression (DE) analysis in ref. 32 Limma performed robust under many conditions. Hence, we selected the gene set using Limma on training set,using a nominal p-value cutoff of 0.05. Finally, we obtained 1854 DE genes.

The DE genes of each pathway were selected as classification features of each base classifier. We took the selected feature sets as input and used SVM based on balanced training data sets (50 pCR vs. 50 RD) to discriminate between the two classes of interest(random sampling 50 RD samples out of 194 RD). For sample type RD, 50 RD were randomly sampled from 194 RD in every run. The performance of each base classifier was tested on training set using 5-fold bootstrap cross-validation with 100 runs (100 × 5). The average accuracy of each base classifier was computed as the final results. The DE pathways were sorted in descending order according to accuracy. Then we selected top 35 DE pathways into *S*.

**Figure 3:**
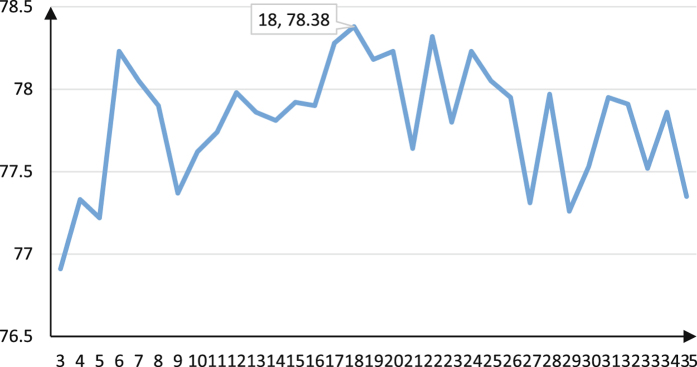
The classification accuracies of ensemble learning with respect to the number of base classifiers based on DE pathways selected from *S* ^*^, ranging from top 3 to top 35.

The pairwise functional diversities between TOP 35 base classifier based on DE pathways were calculated 100 times with each other one. Taking the average, then, a diversity matrix was obtained. According to algorithm 1, we reordered DE pathways by overall diversity of each DE pathway and put it in *S* ^*^. In the case where classifier based on DE pathway was added into ensembles one by one in each iteration step in order from *S* ^*^. Each iteration employed 5-fold bootstrap cross-validation with 100 runs (100 × 5) and the average accuracy of each ensemble classifier was computed as the final results (see Fig. 3). After all iterations were completed, the highest accuracy with 0.7838 was obtained by the ensemble with 18 (*m*) base classifiers.

Then, 9 base classifiers with diversity threshold *θ* less than 0.15 were removed based on algorithm 1 and the remaining 9 base classifiers based on DE pathways were combined for ensemble learning (see Table 2).

**Table 2:** Pathways corresponding to base classifiers of the ensemble in GSE20566 and GSE20194.

Data	Pathway ID	Pathway name	Number of DE/original pathway genes	Drug target
GSE20566	hsa03450	Non-homologous end-joining	4/13	NA
	hsa04750	Inflammatory mediator regulation of TRP channels	10/99	HTR2A HRH1 PRKCE PRKCD PRKCA PRKCB PRKCG PRKCQ
	hsa04060	Cytokine-cytokine receptor interaction	26/265	VEGFA TNFS11 PRLR EGFR
	hsa04360	Axon guidance	16/176	PRKCA
	hsa05168	Herpes simplex infection	21/185	JUN NFKB1
	hsa04310	Wnt signaling pathway	20/144	JUN PRKCA PRKCB PRKCG
	hsa04340	Hedgehog signaling pathway	7/48	BCL2
	hsa04070	Phosphatidylinositol signaling system	15/99	PRKCA PRKCB PRKCG
	hsa05220	Chronic myeloid leukemia	11/73	NFKB1 CDK4 CDK6
GSE20194	hsa00330	Arginine and proline metabolism	3/52	NA
	hsa04974	Protein digestion and absorption	2/90	NA
	hsa04810	Regulation of actin cytoskeleton	7/216	EGFR CHRM1 CHRM2 CHRM3 CHRM4 CHRM5
	hsa05010	Alzheimer’s disease	9/171	MAPT
	hsa00010	Glycolysis/Gluconeogenesis	5/67	NA

Note: NA denotes not found.

**Figure 4:**
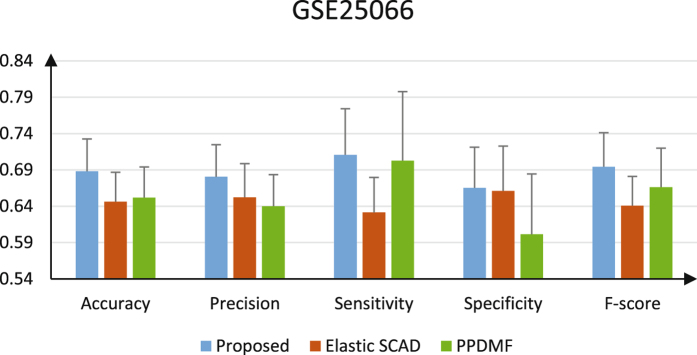
The performance metrics of each method on GSE25066.

In order to form a baseline measure, the performance of ensemble learning classifier with the remaining 9 base classifiers was tested on testing dataset using 5-fold bootstrap cross-validation for 100 runs (100 × 5). Finally, the accuracy, precision, sensitivity, specificity and F-score were computed for each run and then averaged over runs for ensemble classifier. The average accuracy, precision, sensitivity, specificity and F-score of the proposed method are 68.81%, 68.05%, 71.08%, 66.53%, 69.43% on Dataset GSE25066, respectively (see Fig. 4).

### Comparison with other state-of-the-art methods in classification performance

To assess the validity of the proposed approach, here, two latest methods: PPDMF and Elastic SCAD were investigated in parallel with the proposed method on the same public datasets. The PPDMF hypothesizes that pathway-based omics features can provide more information on biological functions for disease diagnosis. This method is a typical representative of pathway-based method for disease diagnosis. It converts omics-level data to pathway-level data by the pathifier algorithm^33, 34^. A pathway dysregulation score matrix in which each score measures the deregulation of a specific pathway for a specific sample is obtained. Then, correlation feature selection (CFS) is used for feature selection. To make this method comparable to our method, the SVM model is used for classification. For Dataset GSE25066, the transcriptomics-level data were firstly transformed to pathway-level data by the pathifier algorithm. Since 3 pathways out of 298 contain only one gene, their dysregulation scores could not be calculated. Hence, we obtained a pathway dysregulation score matrix with 488 rows (samples) and 295 columns (features).

The pathway dysregulation score matrix was also split randomly into two separate matrices according to sample types, half for training (50 pCR vs. 195 RD) and half for testing (49 pCR vs. 194 RD). CFS feature selection was applied with 10-fold cross-validation (10-fold CV) in the training matrix and kept the features that were selected ten out of ten times (100%)^17^. Then, 4 features were selected. A new testing data matrix with 243 rows and 4 columns was generated. Finally, we took the testing data matrix as input and used Support Vector Machine (SVM) to predict patient prognosis. Since dataset GSE25066 is unbalanced between two (RD and pCR) phenotypes in the testing matrix, we balanced the two classes for classification purposes by random sampling 49 samples from the larger collection of testing RD samples. The performance of PPDMF was tested using 5-fold bootstrap cross-validation for 100 runs (100 × 5). Finally, the Accuracy, Precision, Sensitivity, Specificity and F-score were computed for each run and then averaged over runs. The results were obtained with average accuracy of 65.20%, precision of 64.01%, sensitivity of 70.29%, specificity of 60.12%, F-score of 66.64%(see Fig. 4).

The Elastic SCAD method is a typical representative of the regularization method for classification and feature selection tasks. It is a penalty function providing an automatic feature selection for SVM classification tasks combining smoothly clipped absolute deviation penalty (SCAD) and ridge penalties. Elastic SCAD provides robust classifiers in sparse and non-sparse situations. For Dataset GSE25066, also since dataset GSE25066 is unbalanced between two (RD and pCR) phenotypes in training dataset and testing dataset, we balanced the two classes for classification purposes with same process as our method in training dataset and testing dataset. In Elastic SCAD, we set the search interval for both parameters to $[{\lambda }_{l,min},{\lambda }_{l,max}]={\mathrm{[2}}^{-10}\mathrm{,}{2}^{10}],l=1,2$. The procedure of Elastic SCAD repeated 100 times, and then kept the features that were also selected 100 out of 100 times (100%), similarly. The SCAD SVM reduced the number of features from 13236 to 33. Then we took the selected feature sets as input and used SVM to predict disease diagnosis. The performance of Elastic SCAD was tested in the balanced testing dataset using 5-fold bootstrap cross-validation for 100 runs (100 × 5). Finally, the accuracy, precision, sensitivity, specificity and F-score were computed for each run and then averaged over runs for this classification model (see Fig. 4).

Comparing the other two methods over dataset GSE25066, the results show that the proposed method has the higher performances and performed well on all metrics (see Fig. 4), with average accuracy of 68.81% compared with 64.62% in the Elastic SCAD and 65.20% in the PPDMF approach, and so on.

For further evaluation, we tested our proposed method on other datasets: Liver and GSE20194. We also compared our proposed method with other prediction algorithms (Elastic SCAD and PPDMF) following the same evaluation strategy. In the proposed method, we obtained parameter *m* equal to 7 and 10 for Liver and GSE20194, respectively. Finally, the remaining 5 base classifiers were combined for ensemble learning based on algorithm 1 for the two datasets (see Tables 2 and 3).

**Table 3:** Pathways corresponding to base classifiers of the ensemble in Liver dataset.

Data	Pathway ID	Pathway name	Number of DE/original pathway genes	Genes associated with liver
Liver	hsa05410	Hypertrophic cardiomyopathy	26/83	TPM2 TPM3 TPM1 CACNA1C
	hsa04330	Notch signaling pathway	19/48	JAG1
	hsa04512	ECM-receptor interaction	24/82	SDC4 LAMA4 LAMC1 THBS1 ITGA6 TNXB VWF AGRN ITGA7 ITGA1 ITGA4 LAMA5ITGA5 ITGB5 VTN ITGB4 FN1 SDC1
	hsa05414	Dilated cardiomyopathy	25/90	ADRB1
	hsa04115	p53 signaling pathway	39/69	IGFBP3

**Figure 5:**
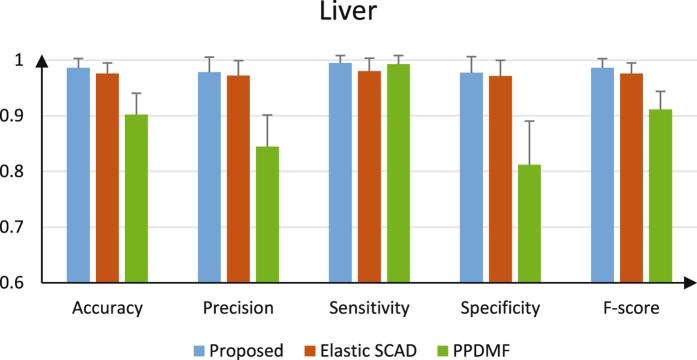
The performance metrics of each method on Liver.

**Figure 6:**
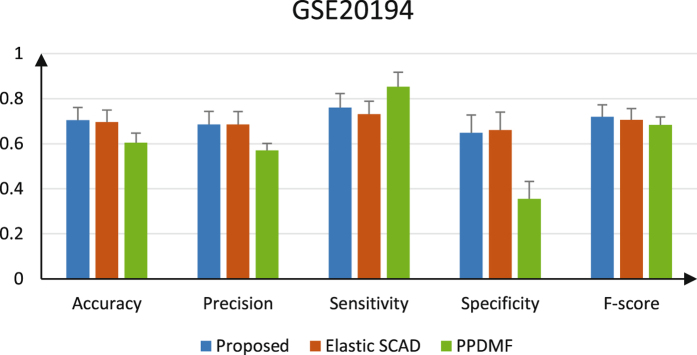
The performance metrics of each method on GSE20194.

Figures 5 and 6 give the comparison of performances for three methods. It is easy to see that the proposed method still shows better performance among most measures as shown in Figs 5 and 6. For GSE20194, the proposed method performs worse than PPDMF and Elastic SCAD only on sensitivity and specificity, respectively. The reason is that the number of features from base classifiers of the ensemble is too few. For GSE25066 dataset, the performances of PPDMF are better than Elastic SCAD, but demonstrate the opposite on Liver dataset. This proofs that the two methods are not robust for different datasets. The reason is that data distributions maybe very different between various platforms. However, the proposed method has the highest performances and perform well on two datasets. Therefore, our algorithm has better robust performance.

### Relationship between identified pathways and drug targets/cancer

In GSE25066, samples are those with diagnosed breast cancer treated with chemotherapy including taxane and anthracycline. Similarly, paclitaxel, 5-fluorouracil, cyclophosphamide and doxorubicin in GSE20194. Response to chemotherapy is categorized as a pathological complete response (pCR) or residual invasive cancer (RD). There are many drugs, which have similar therapeutic mechanisms, for the treatment of breast cancer. To a certain extent, the therapeutic mechanisms of these drugs can reflect the pathogenesis of breast cancer^35^. We believe that the feature genes from the ensemble should be related to the targets of these drugs. In this article, the feature gene set of each base classifier from ensembles was mapped to all breast cancer drug targets, which come from DrugBank (https://www.drugbank.ca/). In GSE25066 and GSE20194, we found that pathways corresponding to base classifiers from ensembles were associated with drugs that are used to treat breast cancer, which reflects the pathogenesis of breast cancer. Then, some clinical breast cancer drug targets were identified in the pathways which were selected into ensembles (see Table 2) in GSE25066 and GSE20194. This illustrates our approach can provide very valuable insights and help in drug target selection, prioritization and validation. In Liver, some genes associated with liver cancer were also identified in the pathways which were selected into ensembles^36^ (see Table 3). Hence, our method can provide clues on potential biomarkers that can suggest novel combinatorial therapies to complex diseases.

### Function annotation of identified module

**Figure 7:**
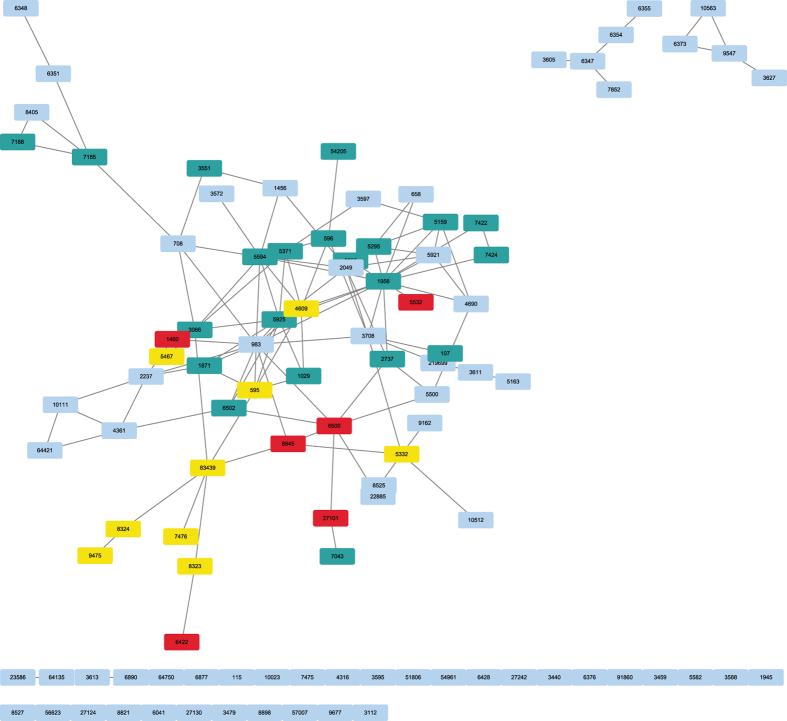
The core biological pathways in network. Blue nodes are a gene set of Pathways in cancer, red nodes are a gene set of Wnt signaling pathway, yellow nodes are common genes for both pathways.

**Figure 8:**
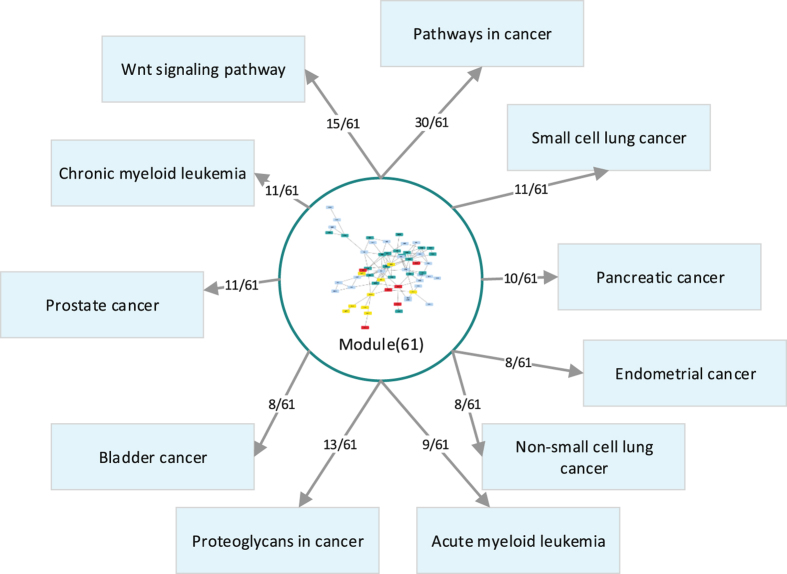
Relationship between the module and other cancer-related pathways.

**Figure 9:**
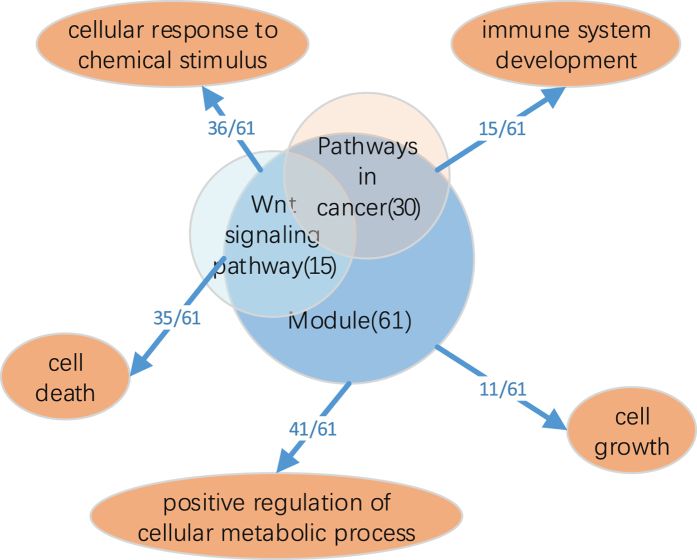
Relationship between the module and biological processes.

To better understand and dissect the complexities of the feature genes from the ensemble model underlying clinically-relevant phenotypes, all feature genes of the ensemble for GSE25066 were mapped on HumanNet^37^ which is an extended gene functional interaction network for Homo sapiens. We find that most of the genes were either directly or indirectly connected to each other, forming some network modules (see Fig. 7). To explore the functionality of the module. Then, the gene list from the largest module was systematically and integratively analyzed using DAVID (https://david.ncifcrf.gov/conversion.jsp?VFROM=NA). This analysis demonstrates the power of the proposed method to identify cancer-related pathways, including Wnt signaling pathway, Pathways in cancer and so on (see Figs 7 and 8). These findings can help scientist understand the disease mechanism and answer specific drug discovery questions, including target prioritization, inhibitor simulations and co-drugging^38^. In addition, through functional annotation clustering, we found that the list of genes was also correlated with biological process of cancer, such as cell death, cell growth, cellular response to chemical stimulus, immune system development and positive regulation of cellular metabolic process (see Fig. 9). Taken together, these analyses demonstrate our approach can identify core biological pathways and biological process underlying clinically-relevant phenotypes, providing the ability to improve tumor classification to reveal more precise prognosis, or to predict response to chemotherapy drugs, driven by models that represent the complexity of the underlying biological activities.

## Discussion

Integrating prior information of biology, like pathways from databases such as KEGG, has recently been proposed to overcome variability of prognostic signatures and improve their prognostic performance^39, 40^ With the rapidly increasing amount of pathway information databases, it enables researcher further opportunities to understand biological mechanisms of cancer and its phenotypes, connectivity of diseases, mechanisms of drug action at molecular level, etc. Now, the combination of pathway information and gene expression profiles is becoming a central branch of research for cancer classification. In this context, we propose a robust ensemble learning paradigm, which incorporates pathway information, to predict cancer classification.

In conclusion, the results obtained in this study show that the proposed method presents the merit of acquisition of more informative from pathways. The method has improved the classification performances of disease status and performs robust both when classifiers are trained on different datasets and within cross-validated single dataset, comparing with PPDMF and Elastic SCAD. In addition, our method can provide clues on potential biomarkers, core biological pathways and processes that can help make true rational design a drug target selection method through the integration of experimental observations with underlying cellular regulation and signaling pathway.
